# Determination of Main Bearing Dynamic Clearance in a Shield Tunneling Machine Through a Broadband PMUT Array with a Decreased Blind Area and High Accuracy

**DOI:** 10.3390/s25134182

**Published:** 2025-07-04

**Authors:** Guoxi Luo, Haoyu Zhang, Delai Liu, Wenyan Li, Min Li, Zhikang Li, Lin Sun, Ping Yang, Ryutaro Maeda, Libo Zhao

**Affiliations:** 1 State Key Laboratory for Manufacturing Systems Engineering, State Industry-Education Integration Center for Medical Innovations, International Joint Laboratory for Micro/Nano Manufacturing and Measurement Technologies, Shaanxi Innovation Center for Special Sensing and Testing Technology in Extreme Environments, Shaanxi Provincial University Engineering Research Center for Micro/Nano Acoustic Devices and Intelligent Systems, Xi’an Jiaotong University, Xi’an 710049, China; luoguoxi@xjtu.edu.cn (G.L.); gsianf@stu.xjtu.edu.cn (H.Z.); liudelai405974229@163.com (D.L.); liwenyan@jl1.cn (W.L.); limin@xjtu.edu.cn (M.L.); sunlin@xjtu.edu.cn (L.S.); ipe@mail.xjtu.edu.cn (P.Y.); maeda2018@xjtu.edu.cn (R.M.); 2 Shandong Laboratory of Advanced Materials and Green Manufacturing at Yantai, Yantai 264000, China; 3 School of Instrument Science and Technology, Xi’an Jiaotong University, Xi’an 710049, China; 4 School of Mechanical Engineering, Xi’an Jiaotong University, Xi’an 710049, China

**Keywords:** ultrasonic rangefinder, PMUTs, broadening of bandwidth, small blind area, monitoring of axial clearance, tunneling machine

## Abstract

Traditional PMUT ultrasonic ranging systems usually possess a large measurement blind area under the integrated transmit–receive mode, dramatically limiting its distance measurement in confined spaces, such as when determining the clearance of large bearing components. Here, a broadband PMUT rangefinder was designed by integrating six types of different cells with adjacent resonant frequencies into an array. Through overlapping and coupling of the bandwidths from the different cells, the proposed PMUTs showed a wide –6 dB fractional bandwidth of 108% in silicon oil. Due to the broadening of bandwidth, the device could obtain the maximum steady state with less excitation (5 cycles versus 14 cycles) and reduce its residual ring-down (ca. 6 μs versus 15 μs) compared with the traditional PMUT array with the same cells, resulting in a small blind area. The pulse–echo ranging experiments demonstrated that the blind area was effectively reduced to 4.4 mm in air or 12.8 mm in silicon oil, and the error was controlled within ±0.3 mm for distance measurements up to 250 mm. In addition, a specific ultrasound signal processing circuit with functions of transmitting, receiving, and processing ultrasonic waves was developed. Combining the processing circuit and PMUT device, the system was applied to determine the axial clearance of the main bearing in a tunneling machine. This work develops broadband PMUTs with a small blind area and high resolution for distance measurement in narrow and confined spaces, opening up a new path for ultrasonic ranging technology.

## 1. Introduction

Owing to the merits of contactless mode, good directionality, transmissibility, and strong anti-interference ability, ultrasonic technology has been widely used in measurement fields, such as distance measurement [1,2,3], sonar [4,5], medical imaging [6,7], nondestructive testing [8,9,10], flow sensing [11], and impurity monitoring [12]. Taking ultrasonic rangefinders as an example, ultrasonic waves can resist influence from ambient light and electromagnetic interference and can penetrate solid objects. Therefore, ultrasonic ranging technology exhibits unique advantages over conventional laser ranging, radio frequency (RF) ranging, and eddy current ranging. Typically, ultrasonic transducers can be divided into piezoelectric and capacitive types [13] to realize the mutual conversion of electrical energy and mechanical energy. Along with the rapid development of Micro Electromechanical System (MEMS) technology, micromachined ultrasonic transducers (MUTs) promise a miniaturized size, lower power consumption, and higher transduction efficiency compared with traditional bulk ultrasonic transducers, thus broadening their possible applications. Typical capacitive MUTs (CMUTs) usually require a high bias voltage of hundreds of volts and a submicrometer-level capacitive gap to activate ultrasonic function [14], which imposes safety concerns and fabrication complexity. In comparison, piezoelectric MUTs (PMUTs) do not require a bias for operation, and the driving voltage can be as low as several volts with good sound pressure and a good signal-to-noise ratio (SNR) [15]. As such, PMUTs can be excellent candidates as ultrasonic rangefinders in miniaturized and mobile scenarios.

Technically, distance measurement based on PMUTs can be achieved in two modes. One is the opposed mode; by mounting two PMUTs at distant targets, the time of ultrasonic wave flight (ToF) can be measured, and thus, the distance can be calculated. Even though this method is effective, the ranging system is complex due to the rigorous requirements of two PMUT device installations and alignments. The other mode is the integrated transmit–receive mode, which involves transmitting and receiving ultrasonic signals with one single PMUT device aimed at the target object. This strategy can effectively reduce the volume and complexity of the measurement system, exhibiting high potential in miniaturized and mobile applications. However, as the reflected echo signal is relatively weak, the measured distance utilized in the integrated transmit–receive mode is always low. To solve this problem, H. Yang et al. [16] reported a PMUT array design that utilized scandium-doped aluminum nitride (AlScN) as its piezoelectric layer for extended long-range detection. With the introduction of scandium, the piezoelectric performance and sound pressure level were enhanced, and the proposed PMUT array improved the system SNR by more than 5 dB even at a distance as far as 6.8 m. In addition to the enhancement of materials, the optimization of structural design also seems to be a feasible methodology for expanding the measurement distance. A. Suresh et al. [17] designed a dual-electrode to isolate the transmit and receive paths within a single PMUT, thereby avoiding issues of frequency mismatch to enhance the reflected echo signal. In this method, only one single PMUT cell can obtain a sensing range of up to 1.5 m. Nevertheless, the reported ultrasonic rangefinder has a minimum detection limit of 0.3 m, denoted as the detection blind area that is common for the integrated transmit–receive mode [2,18]. On the one hand, sufficient excitation with enough cycles is required for the PMUTs to reach their steady state; on the other hand, a long ring-down would be generated after the stop of excitation due to slow energy dissipation. Based on the above analysis, even though most current work has focused on increasing the measurement range, the existence of blind areas in PMUT rangefinder technology still dramatically limits its applications, especially in the fields of dynamic tiny distance measurement inside mechanical equipment, such as when determining the clearance of bearing components.

Herein, a novel PMUT device with a wide bandwidth is proposed by integrating six types of PMUT cells into an array. Through the overlapping of bandwidths from these cells, the bandwidth was broadened, thereby reducing the system damping ratio and simultaneously suppressing the excitation cycles and residual ring-down to decrease the blind area and resolution, endowing its high potential for the measurement of dynamic tiny distances in narrow and enclosed spaces. Furthermore, the fabricated PMUT rangefinder was applied for the determination of the main bearing clearance in a shield tunneling machine. The encapsulation of the PMUT chip and the installation method for the shield tunneling machine were first studied, and combined with the achievement of a driver and echo acquisition circuit, the changes in the clearance and excavation conditions were presented.

## 2. Design and Fabrication

**Figure 1:**
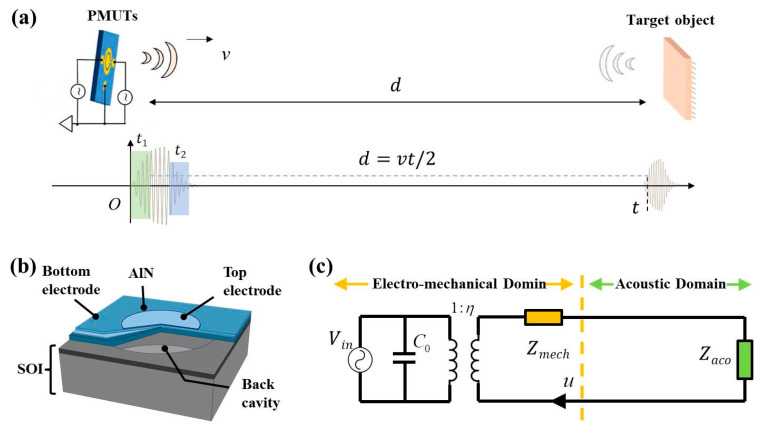
(**a**) The ToF principle for ultrasonic ranging utilizes the integrated transmit–receive mode. (**b**) Schematic structure of a single PMUT cell. (**c**) Equivalent circuit model for a single PMUT cell.

Figure 1a depicts the ToF principle for ultrasonic ranging in the integrated transmit–receive mode. *v* represents the ultrasonic velocity (in air: 340 m/s, in oil: 985.2 m/s), *t* is the ToF, and the distance *d* can be calculated as *d* = *vt*/2. In addition, *t*_1_ and *t*_2_ represent the durations for reaching the steady state and residual ring-down, respectively. The schematic structure of a single PMUT cell is shown in Figure 1b, which follows the traditional unimorph structure composed of an AlN piezoelectric layer sandwiched between the top electrode (Al) and the bottom electrode (doped Si). On the foundation of the piezoelectric mechanism, the cell can generate an ultrasonic wave under an electrical drive and receive an echo signal without external excitation. The equivalent circuit model for one single PMUT cell is established and shown in Figure 1c, in which *V_in_* represents the input excitation, *C*_0_ is the clamp capacitance for the sandwiched structure, *Z_mesh_* is the mechanical impedance of the cell, *η* represents the electromechanical conversion coefficient, *P_in_* is the incident sound pressure, and *Z_aco_* is the acoustic impedance. It can be clearly seen that the model can be equivalent to the coupling of electromechanical–acoustic behaviors.

**Figure 2:**
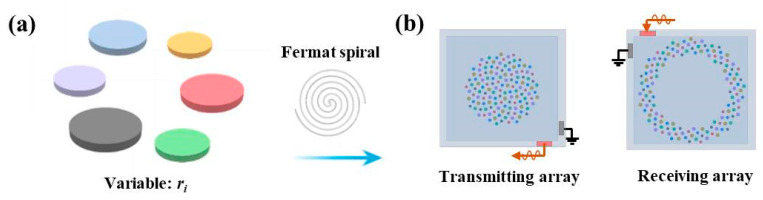
(**a**) Six types of PMUT cells with different diaphragm radii. (**b**) Arrangement of PMUT cells for the transmitting area and receiving area.

According to our previously reported work for modeling PMUT cells and arrays [19], it can be inferred that a smaller systematic mass and higher damping coefficient could lead to a larger fractional bandwidth (FBW). To achieve this goal, a strategy for broadening FBW is proposed through integrating six types of PMUT cells with different diaphragm radii into an array, as shown in Figure 2a. In this way, the FBW could be increased due to the operating bandwidths among different cells overlapping, and thereby, the systematic damping coefficient would be increased to suppress the excitation cycles and residual ring-down. The distribution of PMUT cells for the transmitting area and receiving area is designed following the Fermat spiral arrangement, as shown in Figure 2b, ensuring that the rangefinder works in the integrated transmit–receive mode. Given that the PMUT array devices were fabricated by a typical Multi-user PiezoMUMPs PMUT in MEMScap Inc. (Durham, NC, USA) [20], the thicknesses of the top silicon (*h*_1_) in the silicon on insulation (SOI), piezoelectric layer (*h*_2_), oxide (*h*_3_), and top electrode Al(*h*_4_) were fixed at 10, 0.5, 0.2, and 1 μm, respectively. In this regard, to obtain different resonant frequencies for different PMUT cells, the diaphragm radius of the cell was designed, and Table 1 lists the diaphragm radii for the designed six types of PMUT cells used in this work.

**Figure 3:**
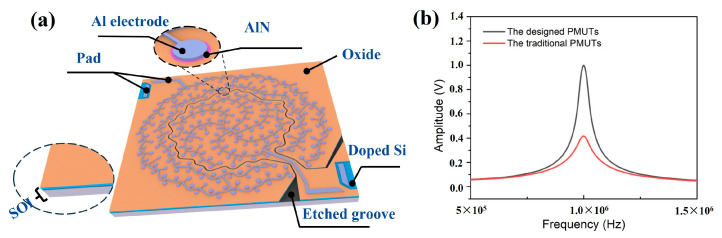
(**a**) The schematic structure of the designed PMUT array device. (**b**) Comparison of the output sound pressures between the designed PMUT device and traditional PMUTs.

Figure 3a exhibits the schematic structure of the designed PMUT device, which combines the transmitting cells and receiving cells into an array with an etched groove. Based on our established model and MATLAB (R2002b) numerical calculation [19], Figure 3b plots a comparison of the output sound pressure between the designed PMUT device and the traditional PMUT array integrated with the same cells utilizing a diaphragm radius of 141.5 μm with a 1 MHz resonant frequency. Even though the output sound pressure of the designed PMUT device is smaller than that of the traditional PMUT array, the introduction of multi-frequency cells induced a higher FBW, and the bandwidth curve was broadened due to the overlapping bandwidths between different types of cells.

**Figure 4:**
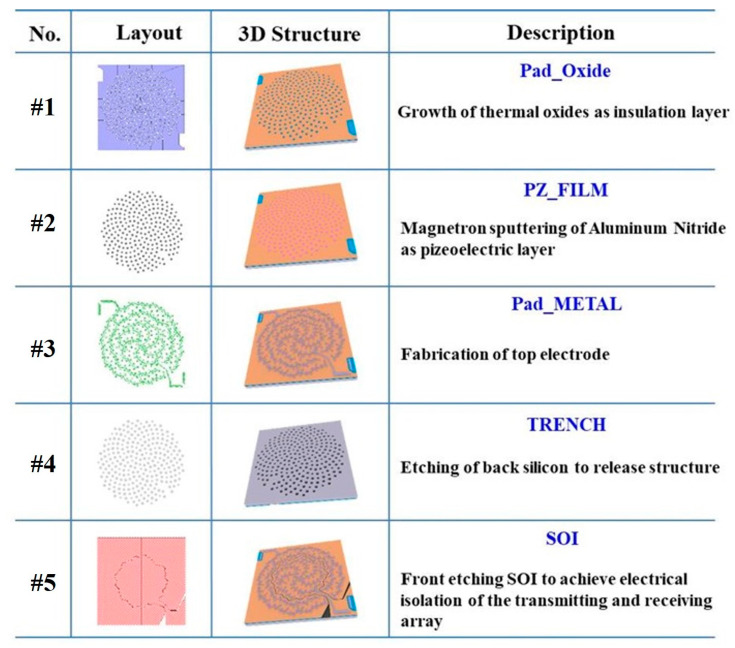
Layout design and fabrication process for the PMUT device.

The designed PMUT array devices were fabricated by a typical Multi-user PiezoMUMPs process, and the layouts were designed as shown in Figure 4. An SOI wafer with a 10 μm silicon device layer and a 0.2 μm oxide layer was utilized for fabrication. First, a layer of phosphosilicate glass (PSG) was deposited on the top silicon layer. Under high-temperature treatment, the phosphorus dopant was driven into the surface of the wafer to act as a bottom electrode for the PMUT cells. In #1 mask fabrication, a thermal oxide with a thickness of 0.2 μm was then grown and etched as the insulation layer. Next, a 0.5 μm AlN was fabricated through magnetron sputtering as the piezoelectric layer, and the AlN was etched and defined as the cell structure in the #2 mask fabrication. Subsequently, a metal layer composed of 1 μm aluminum and 20 nm chromium was deposited, in the #3 mask fabrication, as top electrodes. Then, in #4 mask fabrication, the backside silicon was etched by deep reactive ion etching (DRIE) to release the structure. Finally, the front SOI was etched to achieve electrical isolation of the transmitting and receiving array.

**Figure 5:**
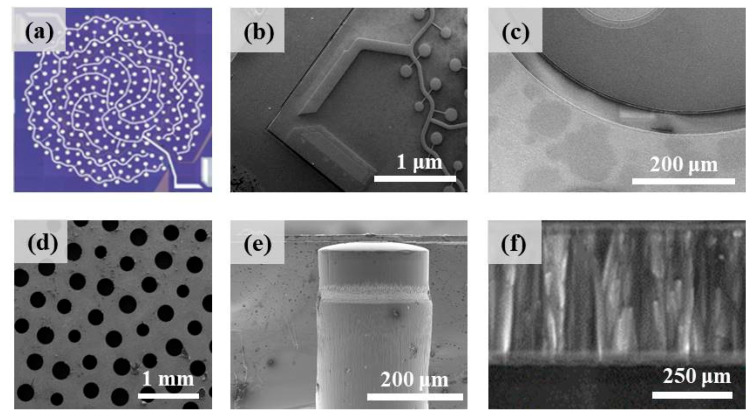
Microscopic observations of the fabricated PMUT device. (**a**) Confocal microscopy image of the top view of this device. (**b**) Top view of PMUT cells and electrical pads. (**c**) Observation of the etched groove to isolate the transmitting and receiving array. (**d**) Backside view of the bottom DRIE openings. (**e**) Cross-sectional view of the etched cavity. (**f**) SEM image of the AlN crystal orientation.

A photo of the diced PMUT chip taken by confocal microscopy is shown in Figure 5a. The concentric inner circular PMUT array for ultrasonic emission and the outer annular PMUT array for receiving ultrasound can be clearly observed. To further evaluate the fabrication quality, scanning electron microscopy (SEM) observations were performed. The images in Figure 5b,c exhibit the top view of the as-prepared device, and the PMUT cells and the etched groove used to isolate the transmitting and receiving array can be clearly seen. The backside of the device is shown in Figure 5d. The bottom DRIE openings were uniformly distributed, and different diaphragm radii for six types of PMUT cells were demonstrated. Figure 5e shows the cross-sectional view of the etched cavity designed with a radius of 110 μm. Due to the possible over-etching and under-etching of the DRIE process, the side wall was not perfectly steep or straight. The as-prepared AlN film is shown in Figure 5f. As we can see, the sputtered AlN thin film has an excellent crystal orientation, as the c-axis of the AlN film is well aligned, indicating good piezoelectric properties [21,22].

## 3. Results and Discussion

**Figure 6:**
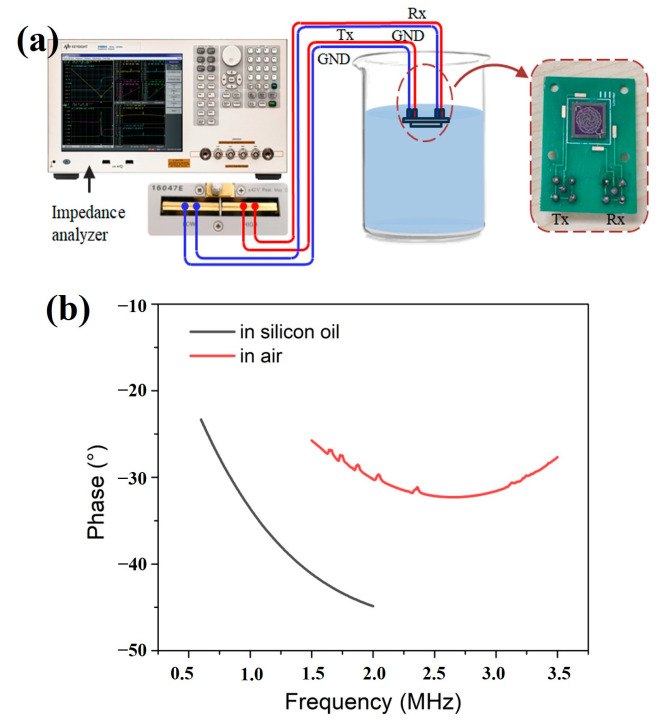
(**a**) The setup for electrical characterization. (**b**) The phase change in silicon oil and air.

To characterize the electrical performance, an impedance analyzer (Keysight, E4990A, Santa Rosa, CA, USA) was utilized to measure the electrical impedance in air and silicon oil, as shown in Figure 6a. Figure 6b exhibits a series of peaks as a result of the integration of six different air-coupled PMUT cells (red line). The six peaks correspond to resonant frequencies of 1.63 M, 1.72 M, 1.86 M, 2.02 M, 2.33 M, and 3.12 M, respectively. After immersion in silicon oil, due to the enhanced acoustic damping of the fluid medium, the resonant frequency decreased, and the frequency response was observed as a flat impedance curve (black line) rather than several narrow peaks, revealing that the bandwidth was broadened through the proposed design. Experimentally, a wide −6 dB FBW of 108% (or −3 dB FBW of 41%) could be calculated from the bandwidth merging curve.

**Figure 7:**
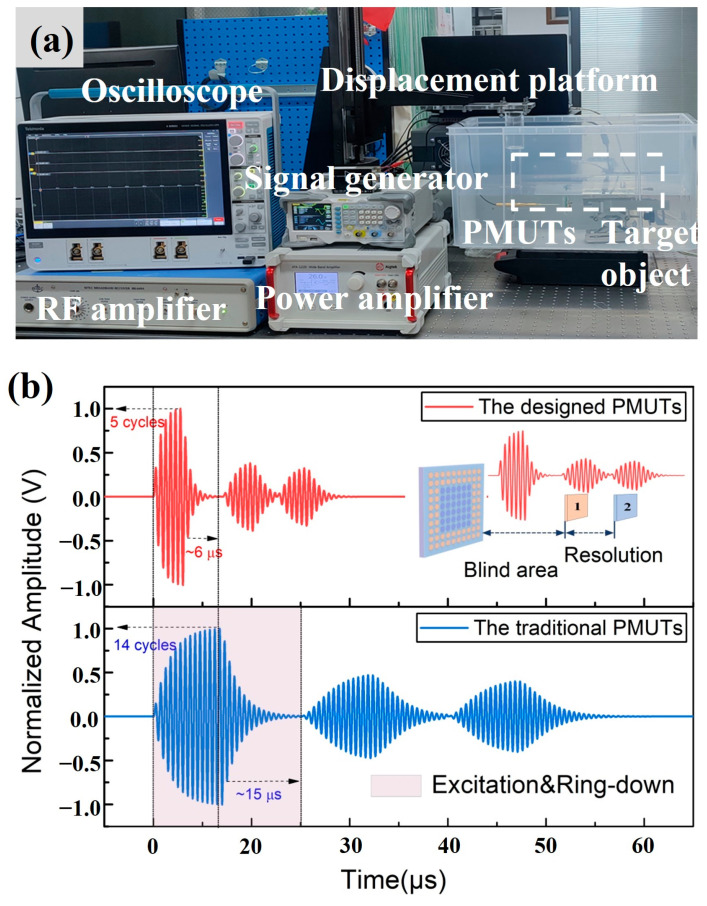
(**a**) The setup for distance measurement. (**b**) Comparison of the designed PMUTs and traditional PMUTs for excitation cycles, residual ring-down, and detection blind areas.

In order to evaluate the rangefinder performance of the designed PMUT device, a custom-made linear displacement platform was established. The platform was based on a precision grating ruler and a linear servo motor to achieve a moving resolution of 0.1 μm and a repeated positioning accuracy of 0.5 μm. The measurement setup is illustrated in Figure 7a with a combination of this displacement platform and a needle hydrophone (Precision Acoustics Ltd., NH-0500, Dorset, UK), which includes a signal generator and a power amplifier to generate the specific extraction voltage for transmitting ultrasonic waves, and the RF amplifier and oscilloscope were used to receive and record the echo signal. Experimentally, a driven signal containing a five-cycle 25 V peak-to-peak sine signal with a frequency of 1000 kHz was applied to completely excite the device. For this measurement, the sampling rate was set to 125 Msps. Furthermore, the time-of-flight (ToF) method based on cross-correlation was utilized for the ranging test. Figure 7b shows a typical excitation and echo signal comparison between the designed PMUT device and the traditional PMUT array with the same diaphragm radius of 141.5 μm. Owing to the broadening of the bandwidth, the designed PMUT device promises a higher systematic damping coefficient, and thereby, fewer excitation cycles (5 cycles versus 14 cycles) and residual ring-down cycles (ca. 6 μs versus 15 μs) were obtained. This corresponds to decreases of over 64.2% and 60%, compared with those of the traditional PMUT array, as shown in Figure 7b. In turn, the detection blind area could decrease dramatically. Based on the measured data and curves, the blind area of the proposed PMUT device could be calculated as ca. 4.4 mm in air or 12.8 mm in silicon oil.

**Figure 8:**
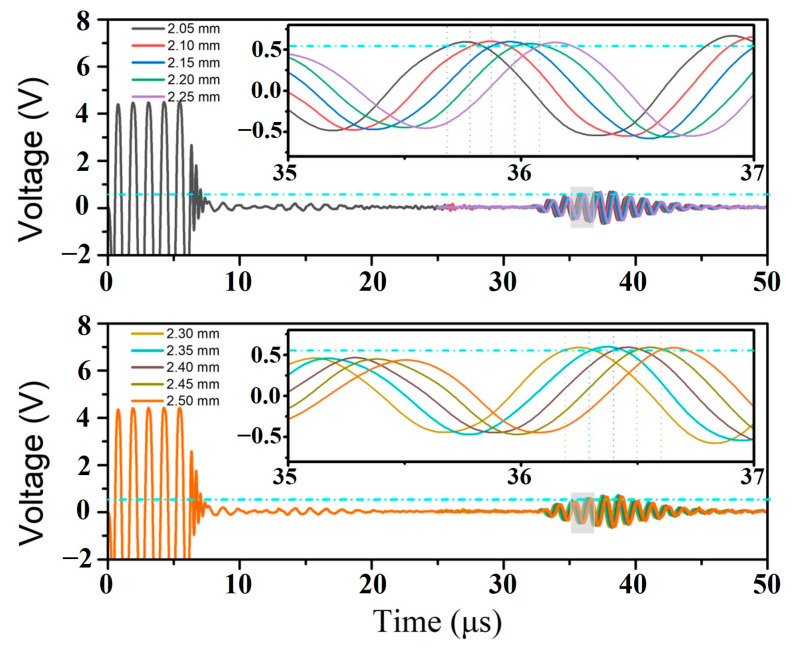
The echo signals for the rangefinder range from 15 to 15.5 mm with a moving step of 50 μm.

Furthermore, the excitation cycles and residual ring-down are dramatically suppressed. Rangefinding for a smaller range of distances could be obtained, highlighting the device’s ability to obtain high-resolution measurements. When the PMUT device was placed at an initial position 15 mm away from the target object and the motor was controlled to move the target baffle away from the PMUT device with a moving step of 50 μm, the measured curves were recorded, as shown in Figure 8, in which the ToF was obtained through an adaptive threshold method and the velocity of the ultrasonic wave was approximately 985.2 m/s at an ambient temperature of 25 °C in silicon oil. From the insets, it can be clearly observed that our PMUT device could distinguish the detection resolution of 50 μm in the measurement range of 15 to 15.5 mm.

**Figure 9:**
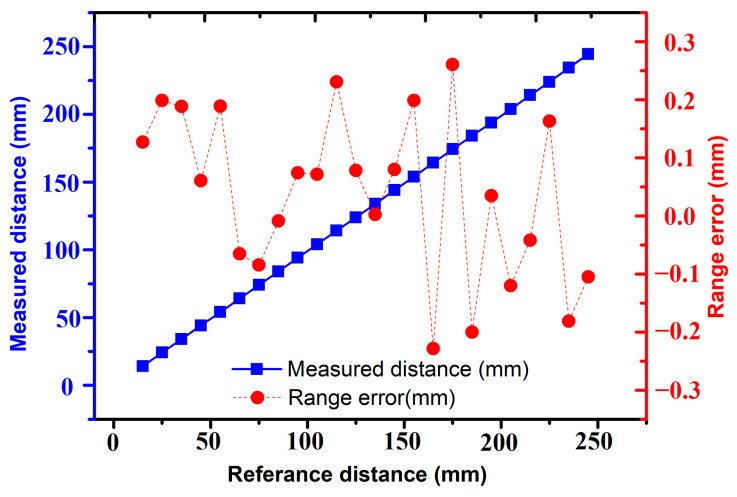
Range error for the distance measurement from 0 to 250 mm.

Apart from the capability of high-resolution detection, the measurement accuracy and range of this device were also investigated. Distance testing was performed as shown in Figure 9, in which the zero position was calibrated by controlling the linear motor to make the target baffle close and stop when it almost came into contact with the PMUT device. Then, the baffle was moved 15 mm away from the device as the starting point of measurement. From this position, the motor moved the target baffle away from the PMUT device with a step of 10 mm. At each measurement point, the waveform was collected 15 times to obtain the mean value. From the results, it could be clearly observed that the measured distances were in good agreement with the reference distance controlled by the precise linear displacement platform. During each step, the range error was well controlled within ±0.3 mm for distance measurements up to 250 mm, corresponding to an excellent 3σ accuracy of 0.445 mm, highlighting the capability of highly accurate measurement under tiny and dynamic distance occasions.

Compared with previously reported methods [1,17,18,23], the proposed PMUT device promises outstanding performance in terms of the blind area, measured distance range, and detection error. The details are shown in Table 2, highlighting the advantages of our PMUTs for dynamic distance measurement in confined spaces.

**Figure 10:**
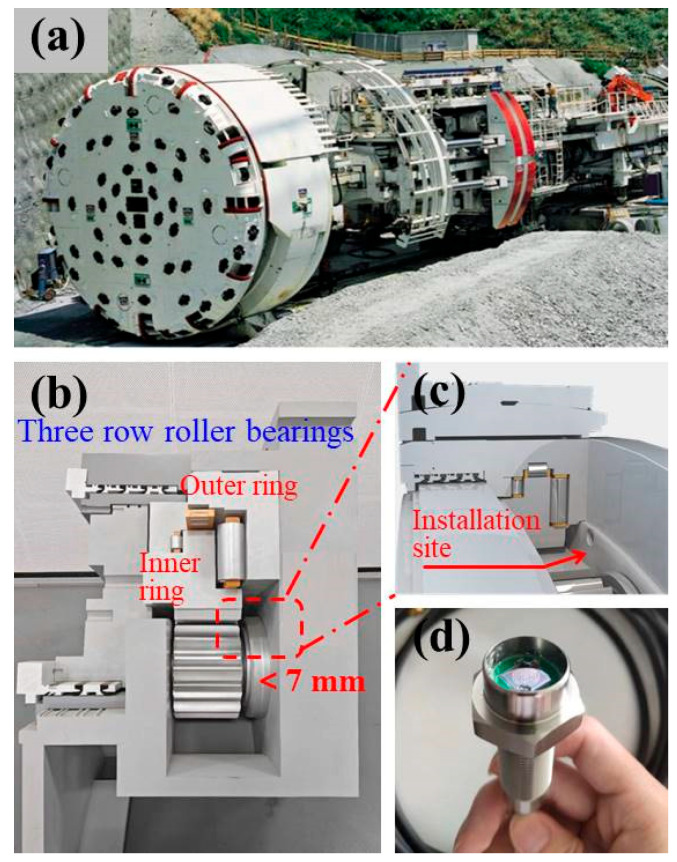
(**a**) The shield tunneling machine. (**b**) Schematic diagram of the internal structure of the main bearing components. (**c**) Installation site. (**d**) The packaged PMUT device.

Owing to their small blind area, high detection resolution, and accuracy, the as-prepared PMUT devices are suitable for tiny and dynamic distance measurements, especially for determining distances in industrial equipment. The shield tunneling machine, as shown in Figure 10a, is a type of large-scale tunneling equipment used in infrastructure construction, and its main bearing axial clearance can reflect the excavation load and construction conditions in real time. However, there is currently no effective monitoring method for the axial clearance of the main bearing of a tunneling machine. Figure 10b depicts the internal structure of the main bearing components in a typical tunneling machine. First, the main bearing of the tunneling machine is located near the drive motor; thus, there is strong electromagnetic interference in the environment, limiting the application of traditional eddy current distance sensors. Second, owing to the confined and narrow measurement space as well as the complex and varied construction conditions, the application of traditional optical ranging methods is also limited. As such, it seems that ultrasonic sensors are suitable for distance measurement in this scenario.

Nevertheless, traditional ultrasonic sensors usually possess a blind area for distance measurement because of the excitation cycles to the steady state and a long residual ring-down. For example, the commonly used commercial ultrasonic ranging chip, *TDK InvenSense CH101*, has a detection blind area that reaches 40 mm. This large detection blind area makes traditional ultrasonic ranging sensors unsuitable for the measurement of the main bearing axial clearance in a shield tunneling machine, as the distance from the fuel tank shell to the inner ring is usually below 10 mm. In this regard, our developed PMUT chip is suitable for the determination of the main bearing axial clearance in a shield tunneling machine. Figure 10c shows the installation site, which is achieved by drilling holes in the outer shell of the fuel tank and targeting the inner ring of the main bearing. The packaged PMUT device is shown in Figure 10d.

**Figure 11:**
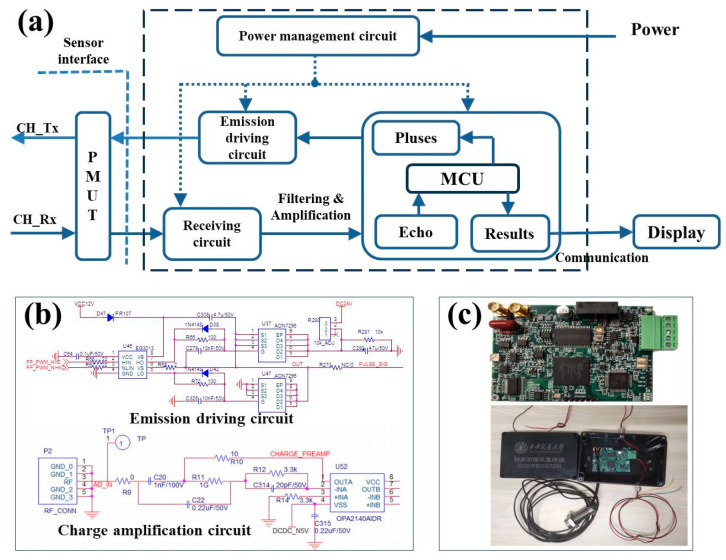
(**a**) The module design for the ultrasonic signal processing circuit. (**b**) Design of the emission driving circuit and charge amplification circuit. (**c**) The as-fabricated circuit module.

In order to meet the practical applications, specialized ultrasonic signal transmission and reception circuits have been developed, as shown in Figure 11a, which include a Microcontroller Unit (MCU), an emission driving module, a receiving module, a power management circuit, and some filtering and amplification modules. First, the power management circuit was driven by a +24 V industrial direct current, and it converts voltages of +3.3 V for driving the MCU and ±5 V for the PMUT device and operational amplifiers. Second, the emission driving module was controlled by the MCU to provide a Pulse Width Modulation (PWM) signal, whose frequency ranges from 500 kHz to 2 MHz to drive the PMUT device to transmit ultrasonic signals. The design of an emission driving circuit is shown in the upper panel of Figure 11b. Third, due to the small amplitude and noise content of the ultrasound echo signals, the ultrasound receiving module includes a preamplifier circuit, a bandpass filter circuit, and an adjustable amplifier circuit. Because of the piezoelectric driving principle of this device, the amplification circuit utilized a charge amplifier module to collect the echo-induced electric signal, and a trap circuit of power interference was designed to reduce noise. Different from traditional ultrasonic echo detection circuits, which utilize a fixed threshold based on a one-time collection curve, here, the waveform is first collected 15 times repeatedly and recorded in the MCU. Then, these data were averaged as a typical echo signal. Through the adoption of this strategy, some noise interference can be eliminated. Moreover, because all the echo curves have been stored, the threshold value can be adjusted in real time instead of using a fixed value. In practical applications, 70% of the peak value of the echo signal was utilized as the threshold to eliminate vibration effects in real excavation conditions.

It is worth mentioning that the sampling rate of the designed echo receiving circuit module reached 100 MHz. This signal processing speed is fast enough to reflect real-time or nearly real-time variation in the clearance of the main bearing as the actual excavation process of the tunneling machine is a low-frequency and high-loading process [24,25]. Finally, the relevant echo curves and calculated distance values were transmitted through the RS485 protocol to the display device or computer. Figure 11c shows the as-fabricated circuit module, which is installed in a water-proof and anti-electromagnetic interference casing. In addition, because temperature changes would impact the speed of ultrasonic waves and thus the ToF, further work will be carried out to develop a temperature compensation algorithm and integrate it into the system to minimize the impact of the environmental temperature.

**Figure 12:**
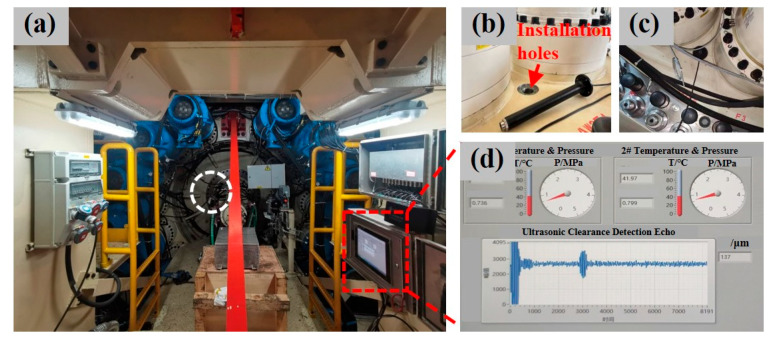
(**a**) Application scenarios of the PMUT device for measuring the axial clearance of the main bearing in the shield tunneling machine. (**b**) The metal sleeve for the package of PMUT devices and the installation site. (**c**) The overall installation situation. (**d**) The monitoring interface of real-time data for temperature, pressure, and axial clearance.

Combining the proposed PMUT sensors and the manufactured signal processing modules, this device can reflect the variation in the dynamic micro-distance and thus determine the axial clearance of the main bearing of a tunneling machine. Figure 12a shows the installation situation in the inner compartment of an earth pressure dual-mode shield tunneling machine, which was fabricated by the China Railway Construction Heavy Industry Group (model: *DL970*). The PMUT sensors were packaged and installed into a metal sleeve, as shown in Figure 12b. The metal sleeve penetrated deep into the main drive box, and the PMUT chip faced the inner ring of the main bearing directly. Figure 12c exhibits a region of the main drive after installation is completed. Furthermore, two temperature–pressure integrated MEMS sensors were also developed and installed into the main drive seal to monitor the variations in temperature and pressure. Finally, a monitoring data interface was developed, as shown in Figure 12d. The detected typical temperature, pressure, and axial clearance ranged from 25 to 60 °C, 0.5–1.2 MPa, and 0–450 μm, respectively, successfully demonstrating real-time monitoring of the axial clearance of the main bearing, temperature, and pressure of the main drive seal.

## 4. Conclusions

In conclusion, a broadband PMUT device was designed through integrating six piezoelectric cells with adjacent resonant frequencies into an array. In this design, the proposed PMUT device promises a broad bandwidth (108% @−6 dB) attributed to the overlapping of each bandwidth from the cells. Due to the wider bandwidth, fewer excitation cycles and shorter ring-down could be achieved, resulting in a small blind area (4.4 mm in air, 12.8 mm in silicon oil) and a high detection resolution (ca. 50 μm) in the integrated transmit–receive mode. The ranging results showed that the PMUT device could achieve a low detection error within ±0.3 mm for distance measurements up to 250 mm. Furthermore, a specialized ultrasonic signal processing module was designed to transmit, receive, store, and process the ultrasonic wave for the PMUT device. In practical applications for monitoring the axial clearance of the main bearing of a tunneling machine, the PMUT chip is packaged into a specific metal sleeve and embedded into the outer shell of the fuel tank to target the inner ring of the main bearing. The actual application results show that this proposed PMUT device can monitor the axial clearance of the main bearing in real time. We envision that the proposed PMUT device would positively impact the fields of dynamic and tiny distance measurement in narrow and confined spaces for status monitoring of industrial equipment.

## Figures and Tables

**Table 1:** The designed six types of diaphragm radii for PMUT cells.

Symbol	Description	Value (μm)
*r_i_*	Radius of diaphragm	110, 126.5, 135.5, 141.5, 147, 151

**Table 2:** Comparison of the main performance parameters.

References	Device	Blind Area/mm	Distance Range/mm	Error/mm
[1]	PMUTs	Not mentioned	100–300	~±0.9
[17]	PMUTs	300	300–1500	Not mentioned
[18]	Bulk	~37	501.9–602.4	±0.202
[23]	PMUTs	~15	15.3–15.5	±0.013
This work	PMUTs	4.4	15–250	±0.3

## Data Availability

The data that support the findings of this study are available within the article.
